# Mechanism of mTOR/RILP-regulated autophagic flux in increased susceptibility to myocardial ischemia-reperfusion in diabetic mice

**DOI:** 10.3389/fphar.2024.1506401

**Published:** 2025-01-31

**Authors:** Jiyao Zhao, Wei Shi, Yan Zheng, Junjie Wang, Muzhao Yuan, Yultuz Anwar, Yuxuan He, Haiping Ma, Jianjiang Wu

**Affiliations:** ^1^ Department of Anesthesiology, The First Affiliated Hospital of Xinjiang Medical University, Urumqi, China; ^2^ Catheterization Laboratory, Changji Prefecture People’s Hospital, Changji Hui Autonomous Prefecture, China

**Keywords:** diabetic cardiomyopathy, autophagic flux, vulnerability, mTOR/RILP pathway, myocardial protection

## Abstract

**Background:**

The increased myocardial vulnerability that occurs in diabetic patients following an ischemia-reperfusion injury (I/RI) represents a significant perioperative safety risk. A comprehensive understanding of the intrinsic mechanisms underlying this phenomenon is therefore of paramount importance.

**Purposes:**

The objective of this study is to investigate the potential mechanism of action between impaired autophagic flux and increased vulnerability in diabetic myocardium. This will provide a foundation for the clinical search for effective preventive and curative measures.

**Methods:**

The transcriptomic alterations in autophagy-related genes following myocardial exposure to I/RI were analyzed by single-cell sequencing. This was followed by the validation of potential mechanisms of action between impaired autophagic flux and increased susceptibility at the cellular and animal levels, respectively.

**Results:**

After I/RI in diabetic myocardium, there was a significant increase in the number of CM1 subgroups and a specific downregulation of 239 autophagy-related genes led by RILP. HE staining revealed that myocardial injury was exacerbated in diabetic mice subjected to I/RI. Transmission electron microscopy revealed that the accumulation of autophagic vesicles in cardiomyocytes of diabetic mice resulted in impaired autophagic flux. qRT-PCR revealed that the expression of RILP was significantly reduced in diabetic mice subjected to I/RI. WB showed that P62 was significantly increased and RILP was significantly decreased in diabetic mice subjected to I/RI compared to healthy mice. Inhibition of mTOR during hypoxia/reoxygenation (H/R) injury restored RILP expression and attenuated cellular injury in cardiomyocytes cultured with high glucose.

**Conclusion:**

Following I/RI in diabetic myocardium, an increase in the CM1 subpopulation and a reduction in RILP expression result in impaired autophagic flux. Regulation of the mTOR/RILP pathway can restore impaired autophagic flux and improve myocardial vulnerability, thereby exerting cardioprotective effects.

## 1 Introduction

The rising prevalence of diabetes worldwide has emerged as a significant public health concern, with the potential to endanger human life and health (GBD, 2023; Heald et al., 2020). Diabetes mellitus not only increases the incidence of cardiac-related complications, but also poses a significant risk to intraoperative safety and postoperative outcomes in such patients (Aniskevich et al., 2017; Liu et al., 2016). Myocardial Ischemia/Reperfusion Injury (I/RI) is one of the most common complications in the perioperative period. Diabetes mellitus has been demonstrated to weaken the heart’s intrinsic protective mechanisms, thereby exacerbating the severity of I/RI (Xu et al., 2019; Ruan et al., 2019). Consequently, a comprehensive understanding of the mechanisms underlying increased vulnerability to I/RI in diabetic myocardium and the development of effective myocardial protection strategies represent significant scientific challenges.

Autophagic flux is a dynamic process comprising the maturation of autophagosomes, transport of autophagosomes and lysosomes, fusion of autophagosomes and lysosomes, degradation of substrates and recirculation of nutrients. The integrity of autophagic flux is of great importance in maintaining cardiovascular homeostasis and function; when autophagic flux is impaired, it leads to impaired cardiac function (Lavandero et al., 2015). Animal experiments have demonstrated that moderate levels of autophagy can counteract myocardial I/RI and attenuate myocardial damage (Kobayashi et al., 2023). However, the changes in autophagic flux after diabetic myocardial I/RI and the potential mechanisms associated with increased myocardial vulnerability remain unclear.

It has been demonstrated that diabetes is associated with impaired cellular autophagy-related functions, and that autophagy plays a role in the various stages of I/RI (Kobayashi et al., 2023). The objective of this study was to analyze the altered transcriptomics of autophagy-related genes after the myocardium was subjected to I/RI by single-cell sequencing, and then to validate them at the cellular and animal levels, respectively (Yu and Dang, 2017; Qiu et al., 2022; Nakai et al., 2007). Furthermore, the study aimed to explore the potential mechanism of action between impaired autophagic flux and increased susceptibility after diabetic myocardial I/RI.

## 2 Materials and methods

### 2.1 Reagents and antibodies

Complete medium (Pricella), sugar-free medium (Solarbio), CCK8 kit (APExBIO), penicillin/streptomycin, foetal bovine serum, trypsin, RILP antibody, P62 antibody, LC3 antibody, mTOR antibody, cell culture box (Shanghai Likang Instrument Co., Ltd.)

### 2.2 Experimental animals, cells and experimental modelling

#### 2.2.1 Ischemia-reperfusion injury model

The study was conducted using eight-week-old C57BL mice and db/db mice (Carvington Laboratory Animals Ltd.). The mice were anaesthetized using isoflurane and mechanically ventilated following tracheal intubation (tidal volume: 30 mL/kg, frequency: 60 beats/min, I:E = 1:2). The thoracic cavity was entered via the third or fourth intercostal space on the left sternal margin. The pericardium was incised, and the heart was exposed. The left anterior descending coronary artery was ligated at a point approximately 2 mm below the root of the left atrium. Successful modelling was demonstrated by the localized pallor of the epicardium and ST-segment elevation. Following a 30-min period of ischemia, the mice were euthanized 120 min after the restoration of the coronary blood supply, and the myocardial tissue was removed.

#### 2.2.2 Hypoxia/reoxygenation model

H9C2 cardiomyocytes (KGI Bio) were cultured in serum-free, sugar-free cell culture medium in an anoxic chamber (95% N₂ and 5% CO₂) for three hours. Thereafter, the cell culture medium was replaced with DMEM medium containing fetal bovine serum, and the cells were cultured for a further three hours in a cell culture incubator (95% O₂ and 5% CO₂, 37°C).

#### 2.2.3 Experimental animals

8–10-week-old male C57 mice and male db/db mice (The genetic background of db/db mice was C57BL/KsJ) were selected respectively (animals were provided by Changzhou Cavinston Laboratory Animal Co. Ltd. in Jiangsu, China).

#### 2.2.4 Grouping of animal experiments

The study was conducted on four groups of mice: (i) C57BL mice that underwent a sham operation (NM), (ii) C57BL mice that underwent an I/RI procedure (NMI), (iii) db/db mice that underwent a sham operation (DM), and (iv) db/db mice that underwent an I/RI procedure (DMI).

#### 2.2.5 Cell experiment grouping and processing

**FIGURE 1:**
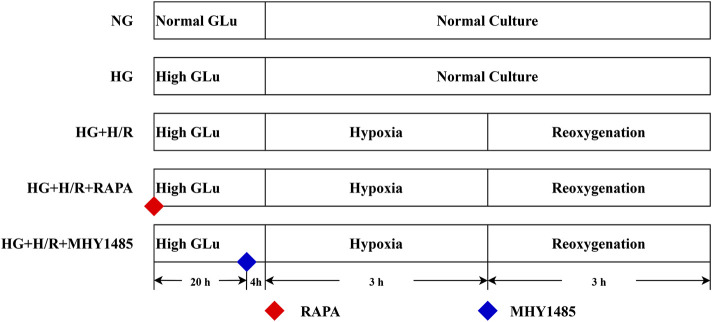
Flowchart of cellular experiments.

(i) Normal glucose (NG) group: H9C2 cells were maintained in a low-glucose medium (5 μM) under continuous culture conditions, without any intervention.
(ii) High-glycemic (HG) group: H9C2 cells were maintained in a continuously culturing environment within a high-glycemic medium (35 μM), without any form of intervention.
(iii) High-glycemic H/R (HG + H/R) group: H9C2 cells were cultured in high-glycemic medium (35 μM) for 24 h and then H/R model modelling was performed.
(iv) RAPA (HG + H/R + RAPA) group: H9C2 cells were cultured in high-glycemic medium (35 μM) and treated with RAPA (10 nM) for 24 h and then H/R model modelling was performed.
(v) MHY1485 (HG + H/R + MHY1485) group: H9C2 cells were cultured in high-glycemic medium (35 μM) and treated with MHY1485 (10 μM) for 24 h and then H/R model modelling was performed (Figure 1).

### 2.3 Experimental indicators and test methods

#### 2.3.1 Single-cell sequencing

After successful modeling according to the experimental groups, the mice were sacrificed by cervical dislocation. Heart tissues were collected and placed in a liquid nitrogen precooled centrifuge tube and frozen in liquid nitrogen for 1–2 h. The heart tissue was pulverized into small fractions of 1 cubic mm, and 10 to 20 pieces of heart tissue were randomly collected to isolate the nuclei. Single-cell RNA sequencing (scRNA-Seq) was conducted on four groups of cardiac tissues using a 10X Genomics Chromium system with an 8-channel microfluidic “double cross” system (Shanghai Bohao Biotechnology Co. Ltd.).

#### 2.3.2 Data preprocessing and normalization

Cell filtration: Cells with total gene count of more than 6,000 and total gene count of less than 200 were filtered out. Gene filtering: Genes expressed in less than 3 cells were filtered, and a count of 1 or more was defined to indicate expression. Other factor filtering: Cells with a 20% greater proportion of mitochondrial genes and cells with more than 1% of hemoglobin genes were filtered out. Genes were classified into 20 interval classes based on the average expression of all genes. The median variance within each interval was calculated, and the absolute value of the variance of the gene mean and the subtraction of the median variance of the corresponding interval median was calculated as the normalized value of the dispersion of the gene.

#### 2.3.3 Cluster analysis and visualization of the data

Louvain algorithm was used to perform cluster analysis on the normalized data. The t-SNE algorithm and UMAP algorithm were used for data visualization.

#### 2.3.4 Marker gene analysis

Marker genes of all clusters were analyzed using the wilcoxon algorithm, and genes that were specifically highly expressed in each Cluster, logFC > 0.25, and expressed in at least 20% of the cells were selected as significant Marker genes for the cluster.

#### 2.3.5 Differential gene analysis

edgeR algorithm was used to analyze the differentially expressed genes. The screening criteria for differentially expressed genes were logFC >1 and P < 0.05.

#### 2.3.6 Analysis of enrichment

KEGG enrichment and GO enrichment were used to analyze the biological functions of differential genes. The functional differences of cell subsets were analyzed by GSEA gene set enrichment algorithm, and the database used was GSEA reactome database.

#### 2.3.7 HE staining

The cardiac tissues of each group were fixed and treated using paraformaldehyde, followed by sequential immersion in xylene and alcohol to deparaffinize. They were then subjected to Harris hematoxylin staining, differentiation, and rehydration before being stained with eosin. After dehydration through sequential immersion in alcohol and xylene, the slices were observed under a microscope and imaged for analysis.

#### 2.3.8 Transmission electron microscopy experiments

The cardiac tissues of each group were fixed and treated with 1% osmium acid, dehydrated by placing them in a series of alcohol solutions with increasing concentrations (30%, 50%, 70%, 80%, 95%, 100%, and 100% alcohol), embedded in resin, polymerized, and sectioned. Subsequently, the samples were stained by placing them sequentially in 2% uranyl acetate saturated alcoholic solution, 2.6% lead citrate solution, and then viewed under the lens of a transmission electron microscope (HITACHI). The images were collected for analysis.

#### 2.3.9 qRT-PCR detection

Total RNA was extracted using Trizol reagent (Thermo Fisher Scientific, United States). Reverse transcription was conducted using 5X All-In-One RT MasterMix (Applied Biological Materials, CAN), while EvaGreen Express 2× qPCR MasterMix-low Rox (Applied Biological Materials, CAN) was employed for reverse transcription and quantitative real-time polymerase chain reaction (qRT-PCR).

#### 2.3.10 Western blot analysis

The total protein was extracted using a RIPA lysate, and the protein concentration was determined using the BCA method. 5× SDS-PAGE loading buffer was added and heated to denature the proteins. Following electrophoresis and membrane transfer, the PVDF membrane was sealed using a sealing solution. The primary antibody was incubated overnight at 4°C (Table 1), rinsed, and the secondary antibody was incubated for 1 h at room temperature. The membrane was then subjected to detection and photography using a ChemiScope mini chemiluminescence instrument. The signals were subsequently detected and quantified using Image Lab 4.0 software.

**TABLE 1:** Antibodies utilized in Western blot analysis.

Primary antibody	Vendor	Catalog	Concentration
β-actin	Sino Biological	100166-MM10	1/2000
MTOR	BOSTER	BM4182	1/2000
RILP	HUABIO	ER61065	1/1000
P62	BOSTER	BM2849	1/1000
LC3	Abcam	AB192890	1/2000

#### 2.3.11 Flow cytometry

The cells in each group were digested with trypsin, the cell precipitate was collected and washed, the cells were resuspended by adding 1× binding buffer and stained sequentially with Annexin V-P and 7-AAD, and flow cytometry (BD) was performed within 30 min.

#### 2.3.12 Determination of cell viability

After treatment according to the experimental grouping, the medium was discarded, 100 ul of 10% CCK-8 solution was added and the cells were incubated in a cell culture incubator for 1 h. The OD value at 450 nm was determined using an enzyme marker and the cell survival rate was calculated.

#### 2.3.13 Statistical analyses

The statistical analyses were conducted using GraphPad Prism 9.5.1 statistical software, and the data are presented as mean ± standard deviation. One-way analysis of variance was used for comparison among multiple groups, Tukey’s test was used for pairwise comparison of one-way means between groups, and Bonferroni correction was used for pairwise comparison of two-way means between groups. Statistical significance was set at P < 0.05.

## 3 Results

### 3.1 In diabetic myocardium, there is a hyperactivation of autophagy and an impaired expression of autophagy-related genes following an I/RI.

**FIGURE 2:**
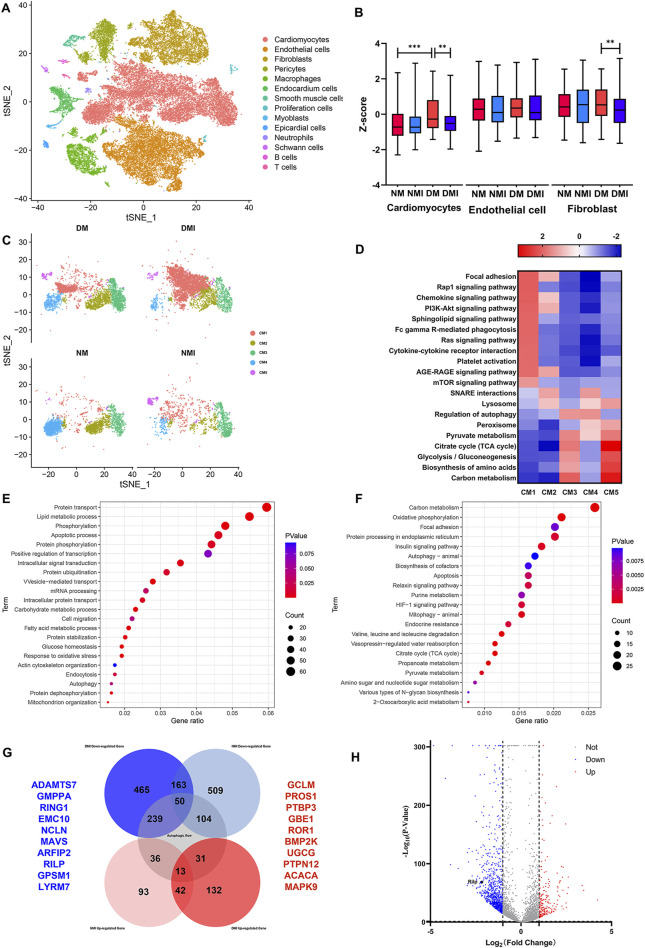
Single cell sequencing results. **(A)** t-SNE mapping of cardiac tissue in each group of mice. **(B)** Box plots of autophagy-related genes by cell subpopulation. **(C)** t-SNE mapping of cardiomyocyte subpopulations in various groups. **(D)** GSEA enrichment analysis of cardiomyocyte subpopulations in various groups. **(E)** GO enrichment analysis of differential genes between DM and DMI groups. **(F)** KEGG enrichment analysis of differential genes between DM and DMI groups. **(G)** Venn diagram of autophagy-related differential genes in normal and diabetic mice undergoing ischemia-reperfusion. **(H)** Volcano plot of differential genes between DM and DMI groups.

The study investigated changes in autophagy-related genes by performing scRNA-Seq on cardiac tissues from NM, NMI, DM and DMI groups. The t-SNE method was used to reduce the data set to its principal components, revealing that the cardiac tissues consisted of 14 cell types. The most dominant cell types were cardiomyocytes, endothelial cells and fibroblasts, which constituted the heart (Figure 2A).

The expression levels of autophagy-related genes (The related genes were obtained from HADb Human Autophagy Database) in different cell types were analyzed, revealing that the expression levels of autophagy-related genes were increased in diabetic cardiomyocytes compared with those in healthy mice. Conversely, the expression levels of autophagy-related genes in cardiomyocytes and fibroblasts of diabetic mice were significantly decreased after I/RI, while the expression of autophagy-related genes in endothelial cells was not significantly affected (Figure 2B). These findings indicate that the expression of autophagy-related genes in cardiomyocytes is more vulnerable to the effects of diabetes and I/RI than in endothelial cells and fibroblasts. It is therefore plausible that aberrant expression of autophagy-related genes may contribute to the increased susceptibility of diabetic myocardium.

The impact of cardiomyocyte heterogeneity on diabetic myocardial vulnerability was subjected to comprehensive analysis. The clustering of cardiomyocytes revealed that the cardiomyocytes were mainly composed of five cellular subpopulations. There was a significant increase in the number of CM1, CM3 and CM5 subpopulations and a significant decrease in the number of CM4 subpopulations in the DM group compared to the NM group. Furthermore, there was an additional increase in the number of CM1 subpopulations and a further decrease in the number of CM4, CM3 and CM5 subpopulations in the DMI group compared to the DM group (Figure 2C; Table.2). Related studies (Liu et al., 2017) have shown that fibroblasts can be reprogrammed into cardiomyocytes. In addition, the proportion of normal cardiomyocytes is reduced after diabetic myocardium is subjected to I/RI, which further leads to an increase in the proportion of CM1 subpopulations. In conclusion, the CM1 subpopulations may have a potential relationship with the formation of diabetic myocardial tissue and I/RI, which should be the focus of further research.

**TABLE 2:** The proportion of each cardiomyocyte subpopulation in total cardiomyocytes.

	CM1	CM2	CM3	CM4	CM5
NM	7.97%	33.47%	15.77%	42.06%	0.73%
NMI	26.80%	14.32%	38.17%	6.79%	13.92%
DM	37.70%	21.14%	24.96%	10.81%	5.38%
DMI	77.05%	10.50%	8.23%	2.22%	1.99%

The GSEA enrichment analysis of the CM1 subpopulation revealed that the PI3K-Akt, RAS, AGE-RAGE and mTOR signaling pathways were significantly enhanced in the CM1 subpopulation in comparison to the other cardiomyocyte subpopulations. Conversely, the lysosomes, autophagy regulation, peroxisomes and the TCA cycle were observed to be weakened (Figure 2D). The Ras signaling pathway has been demonstrated to be closely associated with cardiac hypertrophy and heart failure (Ramos-Kuri et al., 2021). Furthermore, RAGE activation has been shown to increase oxidative stress and trigger inflammatory and fibrotic responses (Wasim et al., 2022). In comparison to other cardiomyocyte subpopulations, the DM1 subpopulation may be dysfunctional in terms of autophagy, energy synthesis, and oxidative stress response. Based on these findings, we propose that the CM1 subpopulation is the causative subpopulation leading to increased vulnerability after I/RI in diabetic myocardium.

A differential gene analysis revealed that there were 209 upregulated genes and 907 downregulated genes in the CM1 subgroup of the DMI group in comparison with the DM group (Figure 2H). A GO enrichment analysis of the differential genes in the DM and DMI groups revealed that the primary functions of these genes were autophagy, the apoptotic process, fatty acid metabolism and the oxidative stress response (Figure 2E). KEGG enrichment analysis of the differential genes revealed that the principal functions were the TCA cycle, mitochondrial autophagy, apoptosis and the HIF-1 signaling pathway (Figure 2F). It was found that the fatty acid metabolism of diabetic mice increased significantly after I/RI, especially the metabolites of very long chain and medium and long chain fatty acids (Ma et al., 2024). This also serves to reinforce the conclusion that autophagy, energy synthesis and oxidative stress are significantly affected in diabetic myocardium subjected to I/RI.

Analysis of differentially expressed genes between healthy myocardium and diabetic myocardium before and after I/RI revealed that 239 autophagy-related genes (The related genes were obtained from GeneCards Database) were specifically downregulated after I/RI in diabetic myocardium, and 31 autophagy-related genes were specifically upregulated after I/RI in diabetic myocardium. In contrast, 104 autophagy-related genes were specifically downregulated and 36 autophagy-related genes were specifically upregulated after I/RI in healthy myocardium (Figure 2G). This again suggests that the expression of autophagy-related genes in diabetic myocardium is susceptible to I/RI. The top 10 downregulated genes were ADAMTS7, GMPPA, RING1, EMC10, NLCN, MAVS, ARFIP2, RILP, GPSM1 and LYRM7. The top 10 specifically upregulated genes were GCLM, PROS1, PTBP3, GBE1, ROR1, BMP2K, UGCG, PTPN12, ACACA and MAPK9(Figure 2H). RILP, as a gene specifically downregulated after diabetic myocardial I/RI, also plays an extremely important role in the transport of autophagosomes. Therefore, we suggest that RILP may be one of the key targets of autophagic flux injury after diabetic myocardial I/RI.

### 3.2 Significant increase in myocardial vulnerability after I/RI in diabetic myocardium

#### 3.2.1 Myocardial vulnerability is significantly increased after I/RI in diabetic myocardium

**FIGURE 3:**
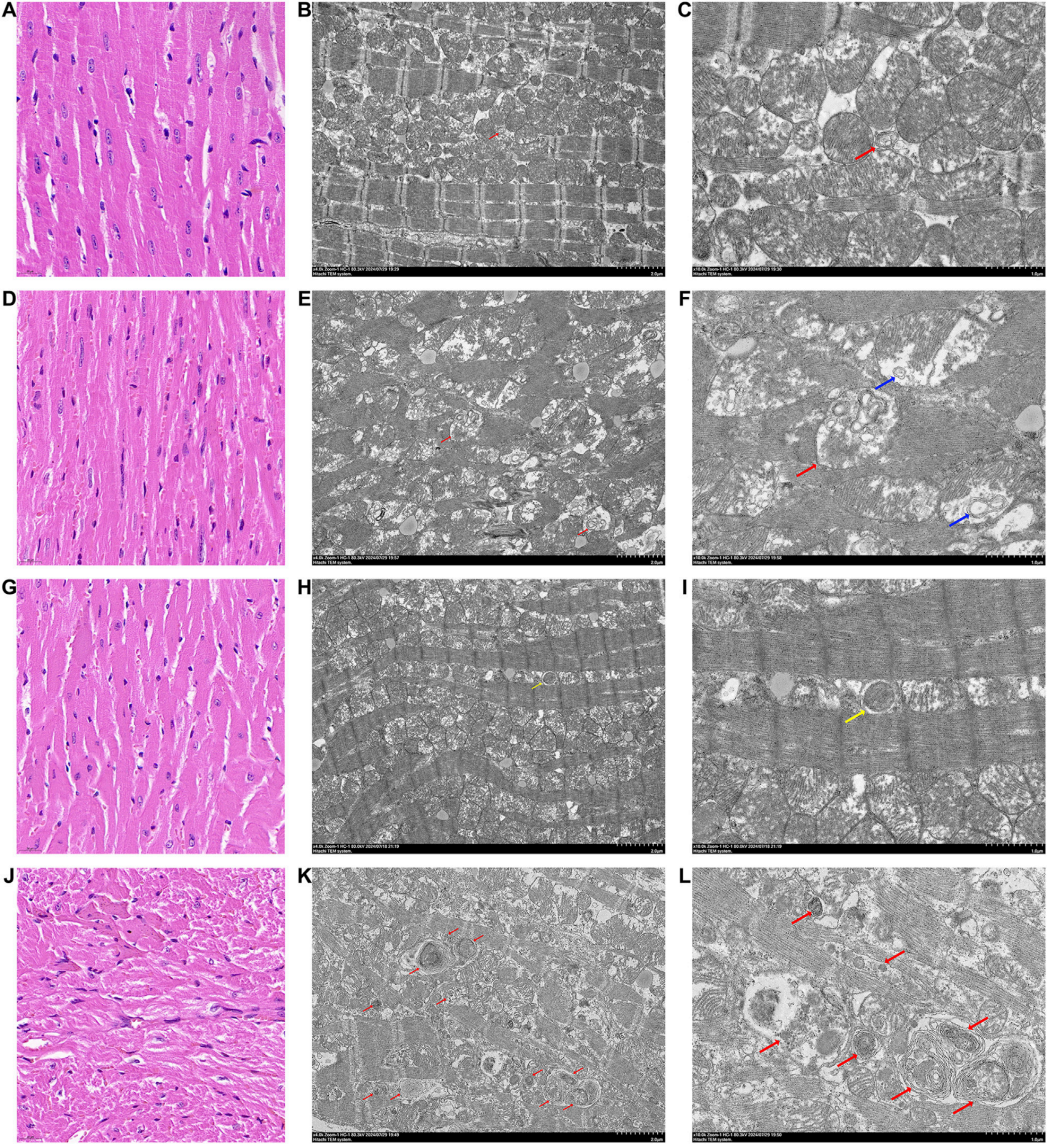
HE staining pictures and TEM pictures of heart tissues of various groups of mice. **(A)** HE staining of heart tissue from mice in the NM group, line segments represent 20 μm. **(B)** TEM picture of heart tissue from mice in the NM group, line segments represent 2 μm. **(C)** TEM picture of heart tissue from mice in the NM group, line segments represent 1 μm. **(D)** HE staining of heart tissue from mice in the NMI group, line segments represent 20 μm. **(E)** TEM picture of heart tissue from mice in the NMI group, line segments represent 2 μm. **(F)** TEM pictures of mouse heart tissue in NMI group, line segments represent 1 μm. **(G)** HE staining of mouse heart tissue in DM group, line segments represent 20 μm. **(H)** TEM pictures of mouse heart tissue in DM group, line segments represent 2 μm. **(I)** TEM pictures of mouse heart tissue in DM group, line segments represent 1 μm. **(J)** HE staining of mouse heart tissue in DMI group, line segments represent 20 μm. **(K)** Electron microscope picture of heart tissue of mice in DMI group, line segments represent 2 μm. **(L)** Electron microscope picture of heart tissue of mice in DMI group, line segments represent 1 μm. (Red arrows are autophagic lysosomes, blue arrows are intra-mitochondrial micro-autophagy, and yellow arrows are autophagic vesicles).

The infarct size of diabetic myocardium increased significantly after I/RI, and the systolic and diastolic function of the heart decreased significantly (Tian et al., 2021). The HE staining of myocardial tissues in mice from each group demonstrated that myocardial cells in the NM group were neatly arranged and exhibited a normal morphology and structure. In contrast, myocardial cells in the DM group displayed disorganization, with heterogeneous cytoplasmic staining, irregular nuclear sizes, and notable infiltration of fibroblasts. In comparison to the NM group, the cardiomyocytes in the NMI group exhibited a notable degree of disorganization, with a considerable number of cells displaying hypertrophy, oedema, degeneration, and evident nucleolysis and nuclear disappearance. In the DMI group, there was a marked increase in myocyte damage and fibroblast infiltration when compared to the NMI group (Figures 3A–J). This suggests that the diabetic myocardium is particularly susceptible to I/RI-induced damage.

#### 3.2.2 The autophagic process is impaired after I/RI in the diabetic myocardium

Transmission electron microscopy observation of cardiac tissues of each group revealed the following: the myocardial tissues of the NM group exhibited no discernible edema of the cytoplasm; myofibrils were neatly arranged, with symmetrically distributed sarcomeres, and the structure of the I-band and A-band was visible, as was that of the Z-line and the H-band; mitochondria did not have obvious edema; and a small amount of autophagic lysosomes was visible (Figures 3B, C).

In the myocardial tissue of the NMI group, myofibrils were observed to be disorganized, with evidence of disruption to the sarcomeres, which were no longer visible. Additionally, the I-band and A-band structures were not discernible, while the Z-line and H-band structures had disappeared. Mitochondria were noted to be moderately swollen, with membranes localized and blurred. The matrix was partially lysed and the cristae were partially broken. Furthermore, autophagic lysosomes were visible in the internal part (Figures 3E, F).

In the myocardial tissue of the DM group, the cytoplasm was not evidently edematous, the arrangement of myofibrils was disordered, sarcomeres were symmetrically distributed, the structure of I-band and A-band was unclear, and the structure of Z-line and the H-band was more ambiguous. Additionally, the mitochondria were slightly swollen, and a small number of autophagic vesicles could be observed (Figures 3H, I).

In the myocardial tissue of the DMI group, the presence of cytoplasmic oedema was observed, accompanied by the fragmentation and lysis of myofibrils in numerous locations. Additionally, sarcomeres were found to be fractured, with the I-band and A-band structures becoming undetectable. The Z-line and H-band structures were also absent, and the rundown disc structure exhibited discontinuity. Mitochondria were observed to be markedly swollen, with blurred membranes in localized areas. The matrix was lysed in localized areas, with cristae exhibiting signs of breakage. Additionally, a small amount of aggregation was noted in specific regions, and a considerable number of autophagic lysosomal structures were visible (Figures 3K, L). The findings indicated that the accumulation of autophagic vesicles and impaired clearance in cardiomyocytes following diabetic myocardium subjected to I/RI resulted in impaire autophagic flux.

#### 3.2.3 The expression of autophagy related gene RILP decreased after I/RI in diabetic myocardium

**FIGURE 4:**
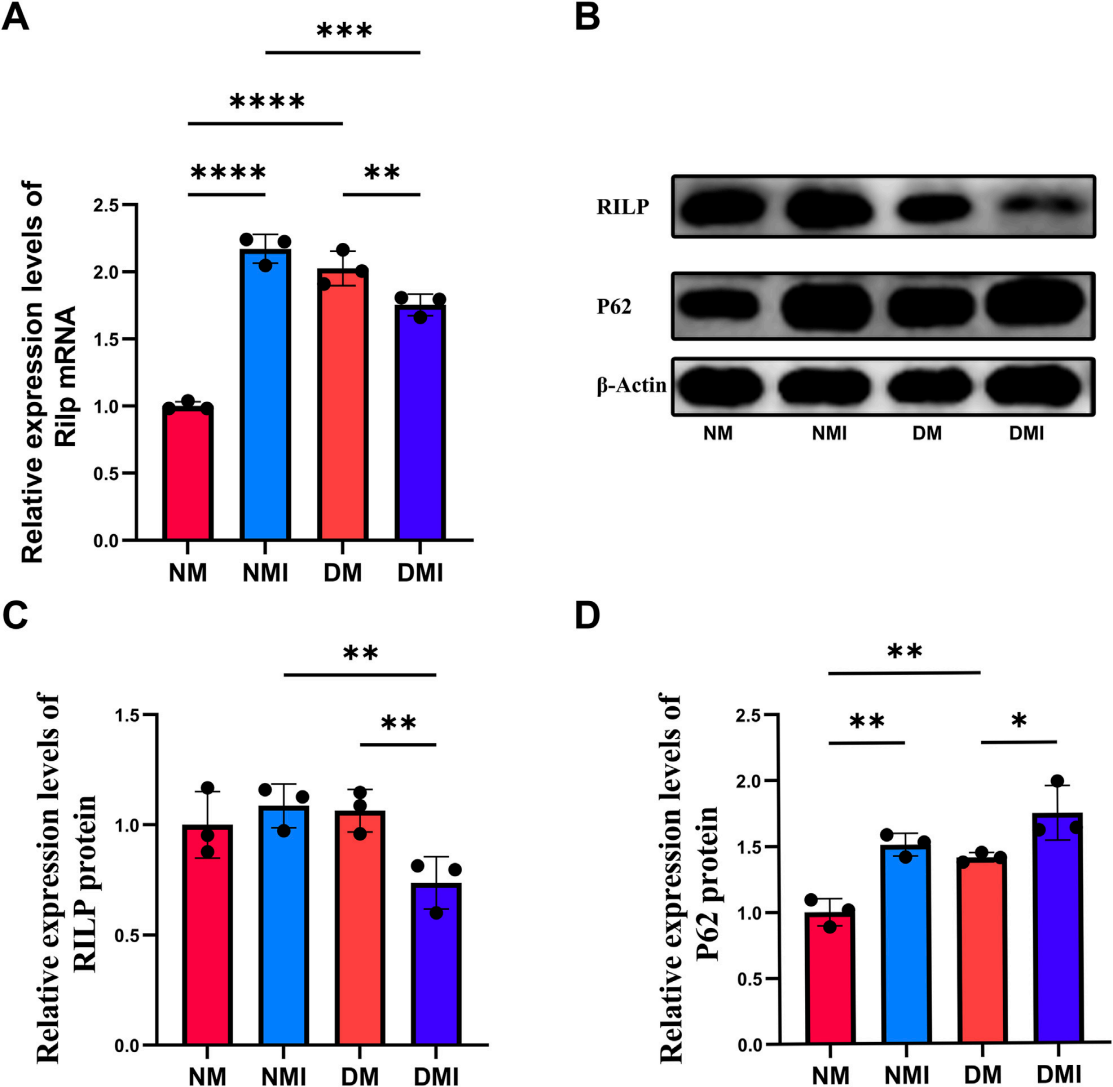
**(A)** q-PCR analysis of Rilp mRNA expression across different groups. **(B)** Immunoblotting results for RILP and P62. **(C)** Relative expression levels of RILP protein. **(D)** Relative expression levels of P62 protein. (*: P < 0.05, **: P < 0.01, ***: P < 0.001, ****: P < 0.0001).

To validate the results of scRNA-Seq, qRT-PCR was used to detect the expression of RILP in cardiac tissue. Compared with the NM group, the transcription level of RILP significantly increased in the NMI group, and also significantly increased in the DM group; compared with the DM group, the transcription level of RILP significantly decreased in the DMI group (Figure 4A). The experiment found that the results are consistent with those of scRNA-Seq, indicating that autophagy is abnormally activated in healthy myocardium after I/RI, and the level of autophagy in diabetic myocardium is also higher than that in healthy myocardium. The decreased transcription level of RILP after diabetic mice suffer from I/RI may lead to abnormal autophagy flux.

#### 3.2.4 RILP protein expression was significantly decreased after I/RI in diabetic myocardium, leading to autophagosome accumulation

P62 is one of the marker proteins reflecting autophagic activity, and its level indirectly reflects the clearance level of autophagic vesicles (Ichimura et al., 2008). When autophagy is activated, P62 protein is continuously degraded in the cytoplasm, and when autophagic activity is weakened and autophagy function is impaired, P62 protein accumulates in the cytoplasm. The expression levels of RILP and P62 in cardiac tissues of each group were detected using WB to reflect the changes in autophagy flux (Figures 4B–D). Compared with the NM group, the expression level of P62 in the DM group significantly increased, indicating that diabetes leads to impaired autophagy function in cardiomyocytes. Compared with the NM group, there was no significant change in the expression of RILP in the NMI group; under normal conditions, I/RI does not affect the expression of RILP. Compared with the DM group, the expression level of P62 significantly increased and the expression level of RILP significantly decreased in the DMI group. Compared with the NMI group, the expression level of RILP in the DMI group significantly decreased. The results indicate that after I/RI occurs in diabetic mice, the expression of myocardial RILP is suppressed, which fails to promote the maturation of late autophagosomes, leading to impaired myocardial autophagy flux and the accumulation of autophagosomes within the cells, resulting in impaired myocardial autophagy flux. The results showed that myocardial RILP expression was inhibited in diabetic mice after the onset of I/RI, which could not promote the maturation of late autophagosomes, resulting in impaired myocardial autophagic flux caused by the accumulation of intracellular autophagosomes.

### 3.3 Autophagic flux damage in cardiomyocytes cultured in high glucose caused by H/R injury can be reversed and cellular damage attenuated by the mTOR/RILP pathway

Abnormal activation of the mTOR pathway in the heart under stress is detrimental and leads to cardiac remodeling and metabolic cardiomyopathy (Sciarretta et al., 2022). Relevant studies have shown that the expression level and spatial localization of RILP are regulated by the mTOR pathway (Khobrekar et al., 2020; Khobrekar and Vallee, 2020).Therefore, we used mTOR inhibitor (RAPA) and mTOR activator (MHY1485) at the cellular level to target mTOR activity and explore their effects on RILP expression, autophagic flux integrity and myocardial protection.

#### 3.3.1 The expression level of RILP was increased by inhibiting mTOR activity

**FIGURE 5:**
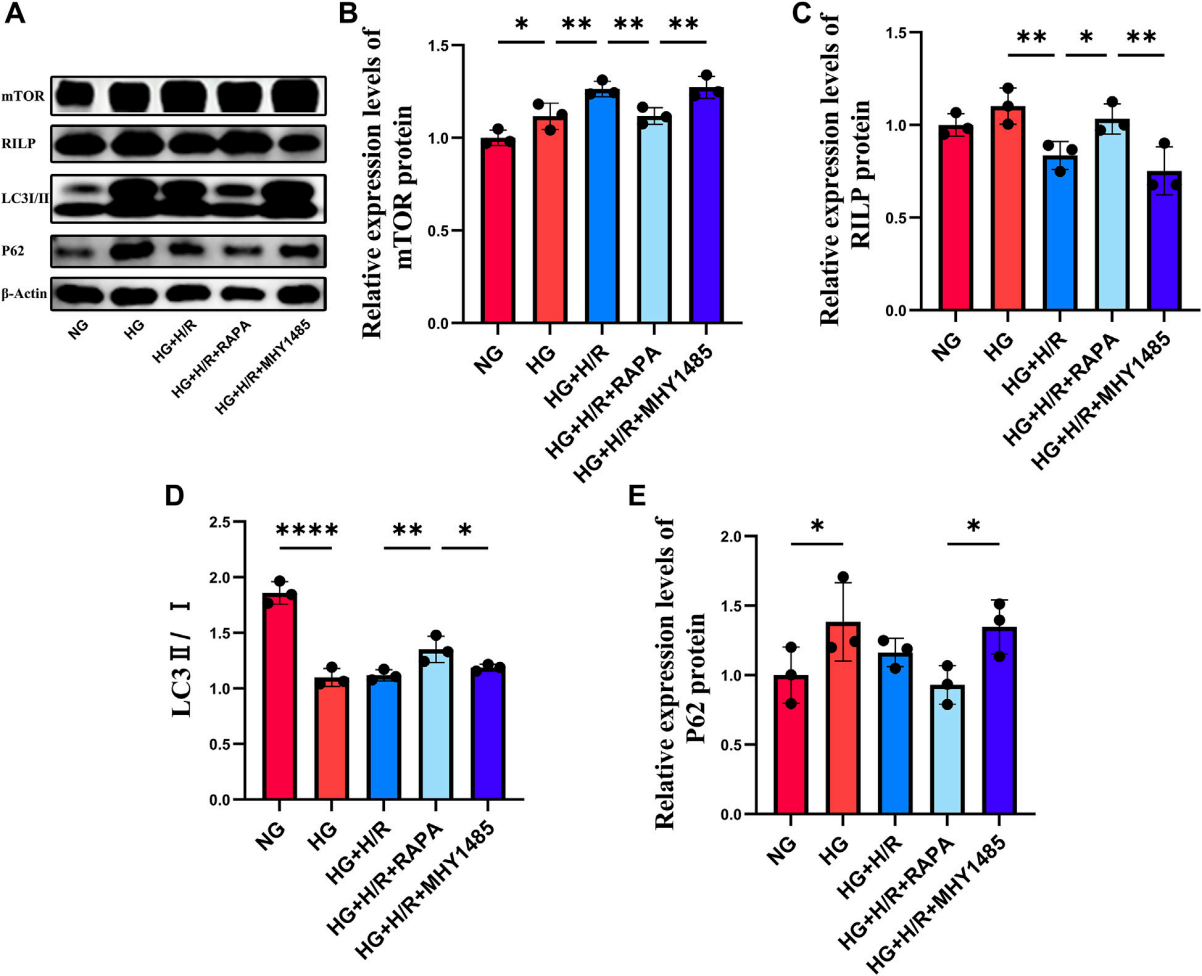
**(A)** WB results for mTOR, RILP, LC3 and P62. **(B)** Relative expression levels of mTOR protein. **(C)** Relative expression levels of RILP protein. **(D)** The ratio of LC3II to LC3I. **(E)** Relative expression levels of P62 protein. (*: P < 0.05, **: P < 0.01, ***: P < 0.001, ****: P < 0.0001).

WB was used to detect the expression of RILP, P62, LC3, and mTOR in cells (Figures 5A–E). Compared with the NG group, there was no significant change in the expression level of RILP in the HG group, while the ratio of LC3II/LC3I was significantly decreased and the expression levels of mTOR and P62 were significantly increased, indicating that a high glucose environment would impair the autophagy function of H9C2 cardiomyocytes. Compared with the HG group, the expression level of mTOR was significantly increased, and the expression of RILP was significantly decreased in the HG + H/R group, indicating that H/R inhibited autophagy and activated the mTOR signaling pathway. Compared with the HG + H/R group, in the HG + H/R + RAPA group, the expression of RILP and the ratio of LC3II/LC3I were significantly increased, and the expression level of mTOR was significantly decreased. Compared with the HG + H/R + RAPA group, in the HG + H/R + MHY1485 group, the expression of RILP and the ratio of LC3II/LC3I were significantly decreased, while the expression of mTOR and P62 were significantly increased. The experimental results showed that H/R injury causing abnormal activation of the mTOR signaling pathway would lead to abnormal expression of RILP and impaired autophagy flux. Inhibition of the mTOR signaling pathway can increase the expression level of RILP and restore the impaired autophagy flux.

#### 3.3.2 Restoration of diabetic myocardial autophagic flux through regulation of mTOR/RILP improves vulnerability

**FIGURE 6:**
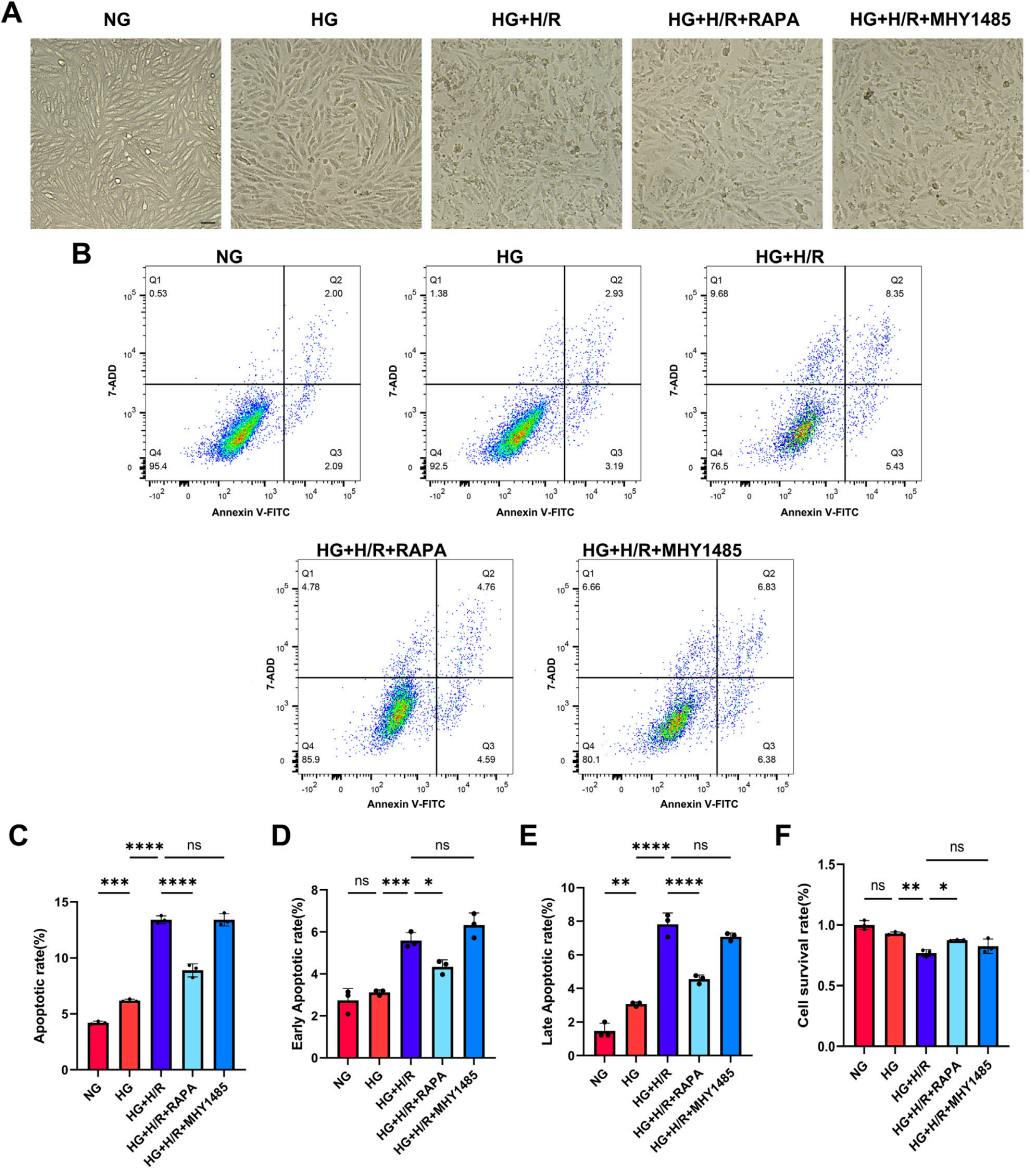
Myocardial cell injury. **(A)** Light micrographs of cardiomyocytes in each group (Bar = 10 um). **(B)** Flow cytometry detection of cell apoptosis in each group. **(C)** Cell apoptosis rate in each group. **(D)** Early apoptosis rate in each group. **(E)** Late apoptosis rate in each group. **(F)** Cell survival rate in each group. (*: P < 0.05,**: P < 0.01,***: P < 0.001,****: P < 0.0001).

Cell morphology was observed by light microscopy and the results showed that the cells in the HG + H/R group were wrinkled compared with those in the HG group, suggesting that H/R caused damage to cardiomyocytes in the high-glucose environment. The morphology of cardiomyocytes in the HG + H/R + RAPA group was improved compared to the HG + H/R group. Compared with the HG + H/R group, the morphology of cardiomyocytes in the HG + H/R + MHY1485 group was not significantly altered (Figure 6A), suggesting that inhibition of mTOR activity was sufficient to attenuate the morphological damage caused by H/R to H9C2 cardiomyocytes in a high glucose environment.

Cell survival and apoptosis were detected by CCK-8 and flow cytometry. Compared with NG group, there was no significant difference in cell survival, but the level of apoptosis and late apoptosis were significantly increased in HG group. Compared with HG group, the survival rate of HG + H/R cells was significantly decreased, and the level of apoptosis, early apoptosis and late apoptosis were significantly increased. Compared with HG + H/R group, the cell survival rate of HG + H/R + RAPA group was significantly increased, the level of apoptosis, early apoptosis and late apoptosis were significantly decreased. Compared with the HG + H/R group, the cell survival rate of the G + H/R + MHY1485 group was significantly decreased, but the level of apoptosis, early apoptosis and late apoptosis had no significant change (Figures 6B–F). The results showed that RILP expression level, mTOR activity inhibition, and recovery of autophagic flow in cardiomyocytes cultured with high glucose after H/R can reduce the level of apoptosis and improve myocardial susceptibility, thus playing a protective role in myocardium.

## 4 Discussion

In this study, we employed a gene transcriptomics to investigate the potential mechanism of action between impaired autophagic flux and increased diabetic myocardial susceptibility. To this end, we examined autophagic flux in healthy and diabetic myocardium after I/RI. Firstly, using by scRNA-Seq, it was demonstrated that diabetes and I/RI result in the abnormal expression of autophagy-related genes in a subpopulation of cardiomyocytes. Of particular significance were the changes observed in RILP, which led to impaired autophagic flux in diabetic cardiomyocytes. Furthermore, the results of the scRNA-Seq and the alterations in autophagic flux were corroborated by animal experiments. The diminished expression of the autophagy-related protein RILP following I/RI in diabetic myocardium resulted in impaired autophagic flux, leading to the accumulation of autophagic vesicles within the cells. Ultimately, the restoration of impaired autophagic flux caused by H/R in high-glucose cultured cardiomyocytes was achieved by inhibiting mTOR activity and increasing RILP expression, thereby providing myocardial protection.

Energy metabolism represents the material basis of all life activities. Impairment of intracellular energy metabolism can result in cellular damage and affect the normal life activities of the organism. Abnormalities in energy metabolism, caused by diabetes mellitus and excessive production of free radicals due to the accumulation of metabolic wastes caused by abnormal autophagy, have been demonstrated to be the primary causative factors in the development of diabetic cardiomyopathy (Dewanjee and Bhattacharjee, 2018; Jia et al., 2018). In the initial stages of ischaemia, autophagy serves to replenish the energy metabolic supply of cardiomyocytes, thereby attenuating myocardial injury (Yu and Dang, 2017; Qiu et al., 2022). During the reperfusion phase, excessive autophagy activation has been demonstrated to affect mitochondrial function, thereby exacerbating myocardial injury (Nakai et al., 2007). It has been demonstrated in existing studies that myocardial injury resulting from I/RI can be mitigated and the restoration of myocardial function can be facilitated by the restoration of impaired autophagic flux (Kubli and Gustafsson, 2014; Gottlieb et al., 2015; Matsui et al., 2007). The present study verified the impaired autophagic flux in diabetic myocardium by gene transcriptomics and proteomics. It was found that RILP-mediated formation of late autophagic lysosomes may be an important cause of the significant increase in diabetic myocardial vulnerability.

Cellular autophagy is the process by which damaged, denatured, or senescent proteins and organelles are transported from the cell to the lysosome for degradation and concomitant recycling of energy substances (Levine and Kroemer, 2019). Autophagy is vital for maintaining cardiac homeostasis under physiological conditions. Conversely, the moderate activation of autophagy in response to stressors such as ischaemia and hypoxia can provide cardiomyocytes with the necessary metabolic substrates, reduce the accumulation of metabolic wastes, and mitigate the extent of myocardial injury. The term “autophagic flux” is used to describe the intensity of the autophagic process occurring within a specific time frame. This is primarily associated with the formation of autophagic vesicles and the maturation of autophagic lysosomes. A number of studies have demonstrated that a range of diseases can result from impaired autophagosome maturation, including neurodegenerative diseases, cancer, and muscle diseases (Zhao and Zhang, 2019; Jiang and Mizushima, 2014). The maturation of autophagosomes is subject to strict regulation and is highly susceptible to fluctuations in energy supply and stress response (Zhao and Zhang, 2019). Consequently, compromised autophagic flux integrity resulting from I/RI in the myocardium of diabetic patients contributes to the heightened vulnerability of diabetic myocardium.

The excessive intracellular accumulation of autophagosomes has been demonstrated to cause cellular damage when the integrity of the autophagic stream is impaired (Bosc et al., 2020). This is evidenced by the accumulation of large amounts of autophagosomes in cardiomyocytes, which has been shown to cause oxidative stress damage and affect normal mitochondrial function (Kanamori et al., 2015; Liang et al., 2015). It has been demonstrated that the hyperactivation of Beclin1 at the reperfusion stage impedes the fusion of autophagosomes with lysosomes, resulting in the disruption of autophagic flux and ultimately leading to cell death (Hein et al., 2003). It is therefore proposed that restoration of the autophagic flux in diabetic myocardium may exert cardioprotective effects by promoting fusion of autophagosomes with lysosomes following the occurrence of I/RI.

Rab Interacting Lysosomal Protein (RILP) contains an α-helical coiled-coil and is involved in a range of physiological processes, including autophagosome biogenesis, transport and degradation. RILP interacts with activated Rab7 through its carboxy-terminal region, thereby driving late endosomal/lysosomal translocation (Khobrekar and Vallee, 2020; Amaya et al., 2016). Inhibition of RILP expression results in impaired integrity of the autophagic flux in neuronal cells (Khobrekar et al., 2020), whereas promotion of RILP expression contributes to the treatment of neurodegenerative diseases (Jiang and Mizushima, 2014). In hepatocellular carcinoma, Rab7-RILP-regulated lysosomal transport represents a crucial determinant of cancer cell invasive capacity (Qi et al., 2022). RILP, as a pivotal element of the autophagic flux, exerts regulatory influence over autophagy through the PI3K/AKT/mTOR signaling pathway (Wei et al., 2023). It may therefore be posited that by targeting and regulating RILP, it could prove beneficial in restoring the impaired integrity of autophagic flux in diabetic myocardium due to I/RI, thereby achieving myocardial protection.

This study has the following limitations: H9C2 cardiomyocytes were used instead of primary cardiomyocytes; This study was only carried out at the cell level and not verified at the animal level. In this study, we investigated the role of mTOR/RILP signaling pathway in diabetic I/RI by using mTOR activators and inhibitors rather than RILP regulation.

In conclusion, the mTOR/RILP signaling pathway represents an intrinsic mechanism for regulating the integrity of autophagic flux. The restoration of autophagic flux in diabetic myocardium through the regulation of the mTOR/RILP signaling pathway may confer cardioprotective benefits, particularly in the context of I/RI-induced vulnerability. It is hoped that the role of the mTOR/RILP signaling pathway in the diabetic myocardium in the context of I/RI will continue to be the subject of further in-depth exploration, with a view to establishing the foundations for the future clinical application of this knowledge.

## Data Availability

The relevant single-cell sequencing data are available from a repository, namely National Center for Biotechnology Information, and can be accessed through the following link: ID 1209376 - BioProject - NCBI.
