# Supplementary figures and images for: Mechanism of mTOR/RILP-regulated autophagic flux in increased susceptibility to myocardial ischemia-reperfusion in diabetic mice
Source: Front Pharmacol. 2025 Jan 31;15:1506401. doi: 10.3389/fphar.2024.1506401 (PMC11825452; doi:10.3389/fphar.2024.1506401)

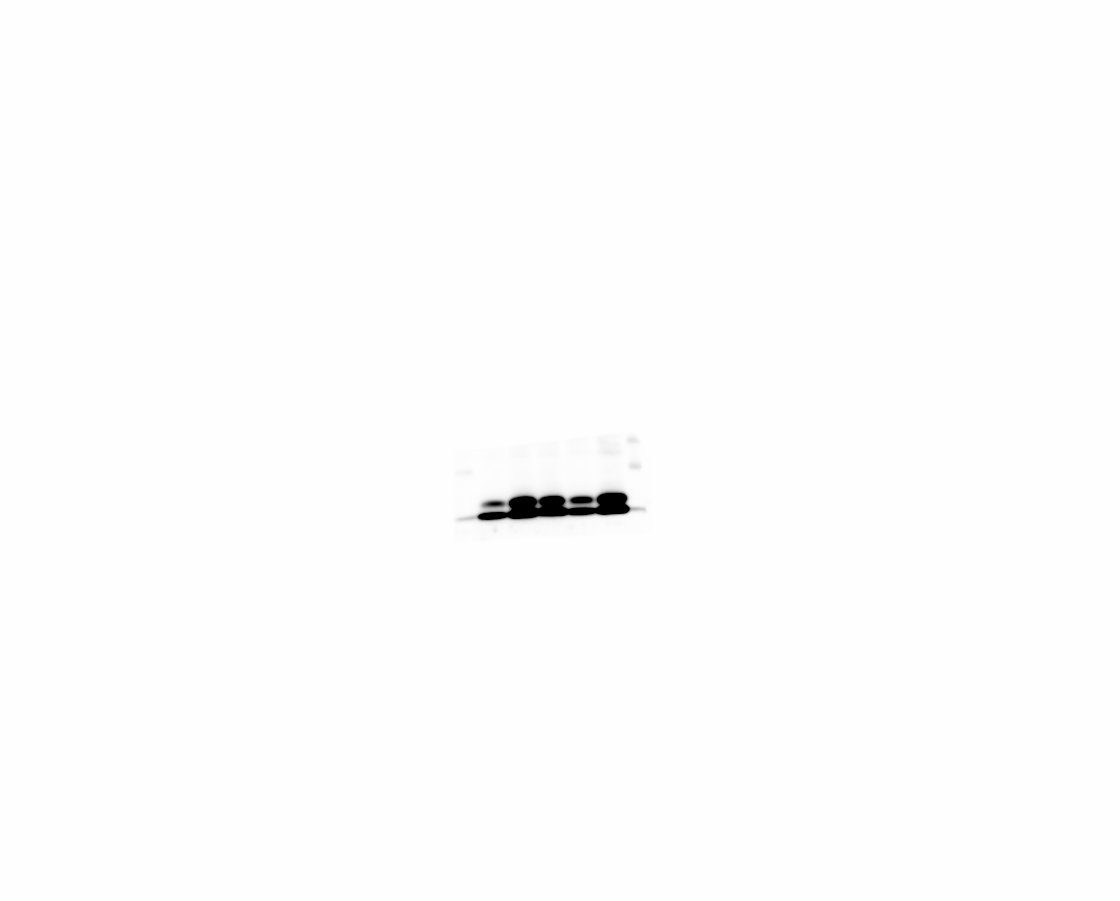

Supplement: Supplementary file 1 [file DataSheet1.zip › WB/LC3-5(1).png]

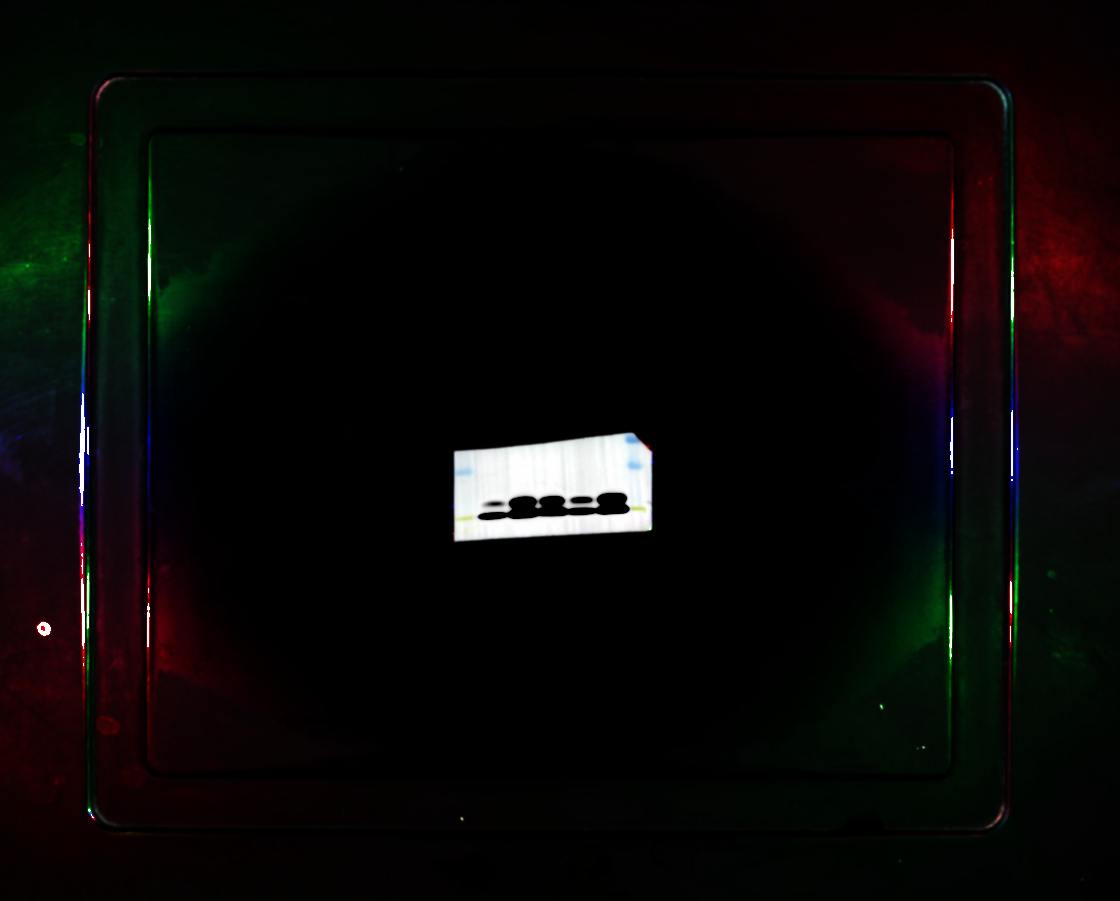

Supplement: Supplementary file 1 [file DataSheet1.zip › WB/LC3-5(2).png]

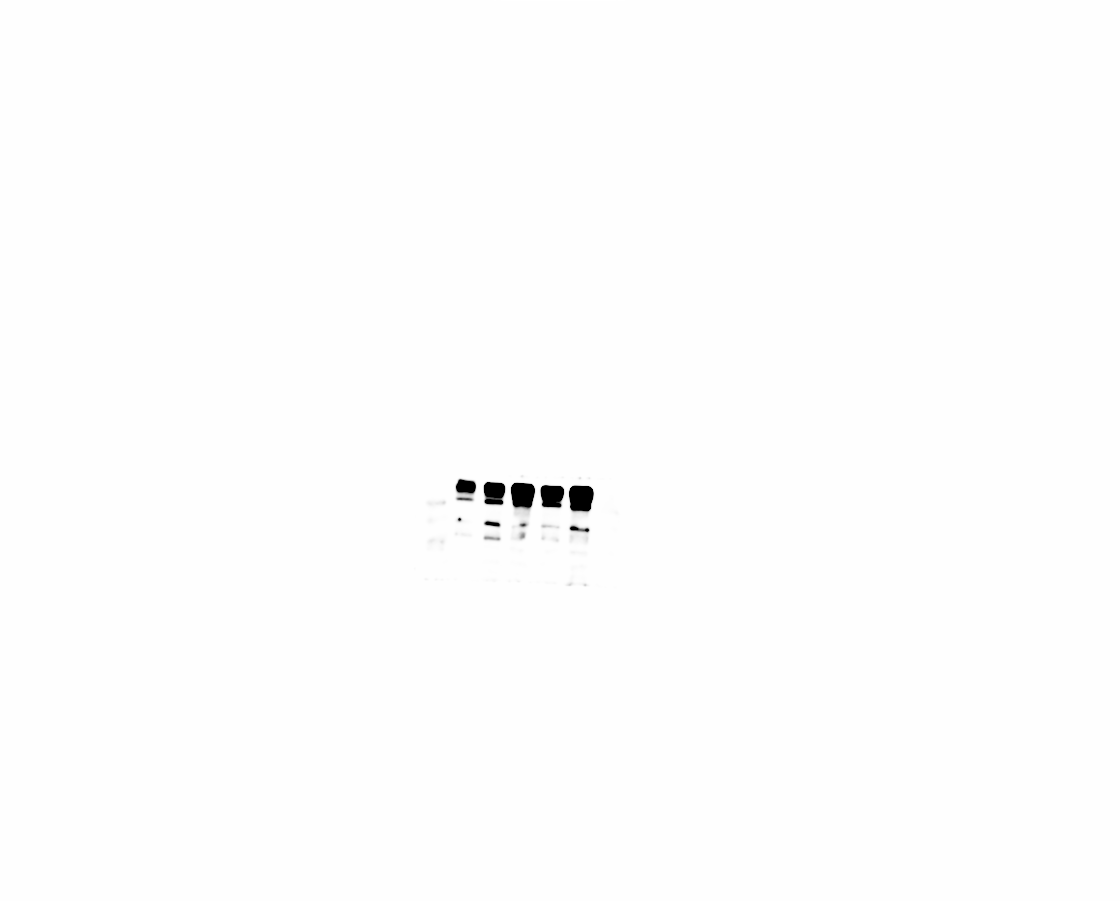

Supplement: Supplementary file 1 [file DataSheet1.zip › WB/mTOR-5(1).tif]

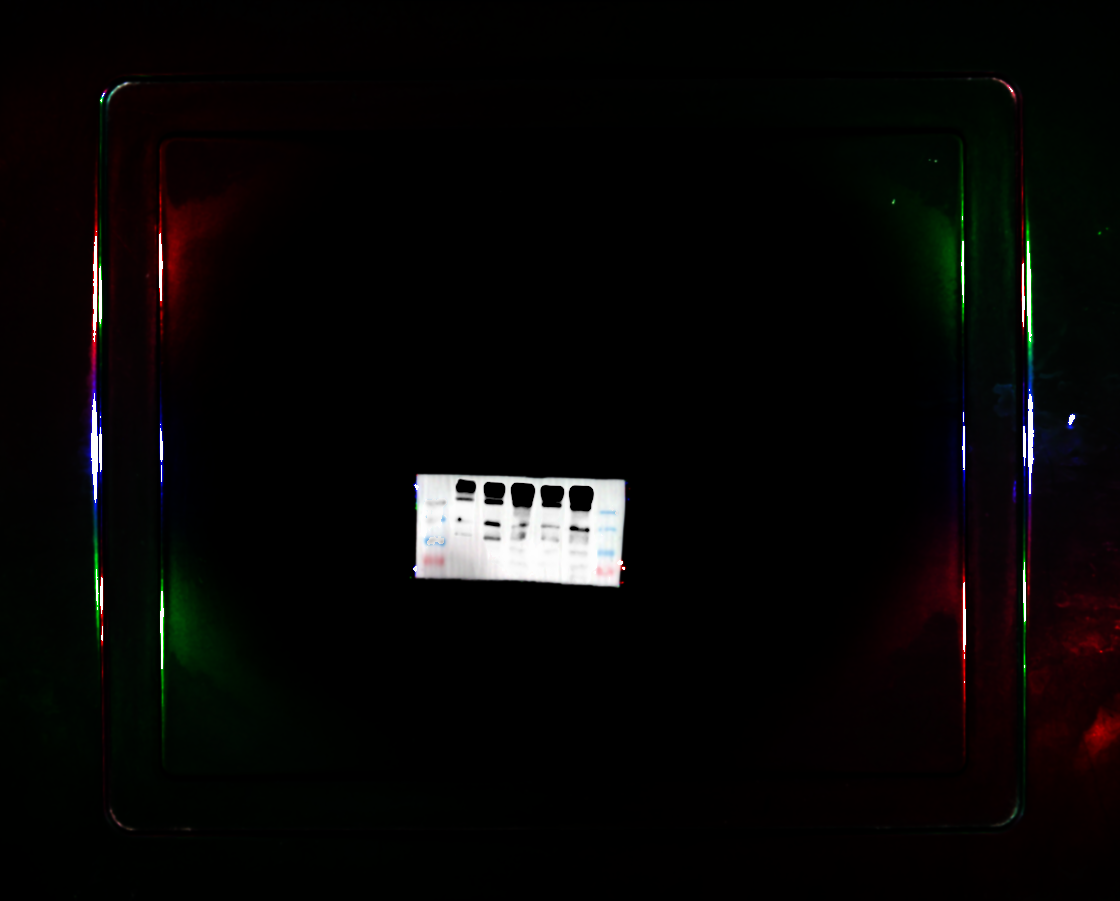

Supplement: Supplementary file 1 [file DataSheet1.zip › WB/mTOR-5(2).tif]

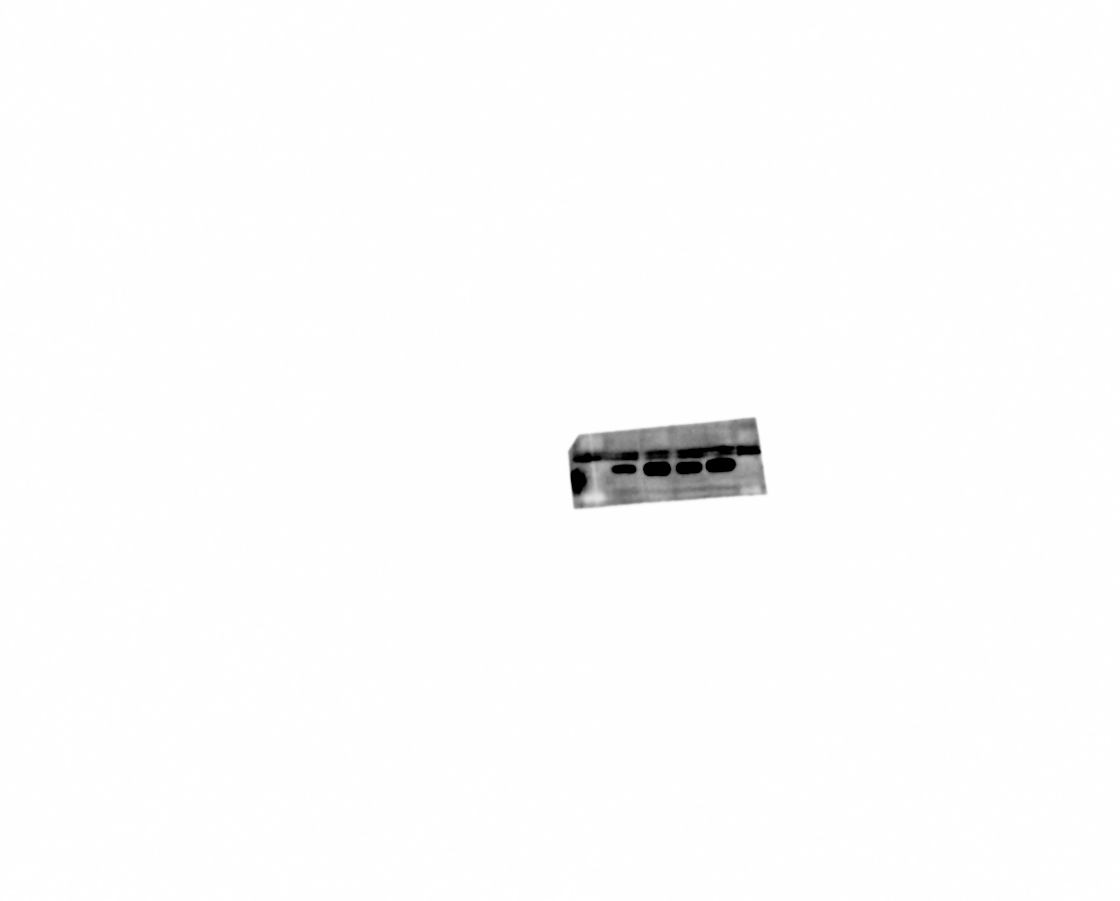

Supplement: Supplementary file 1 [file DataSheet1.zip › WB/P62-4(1).tif]

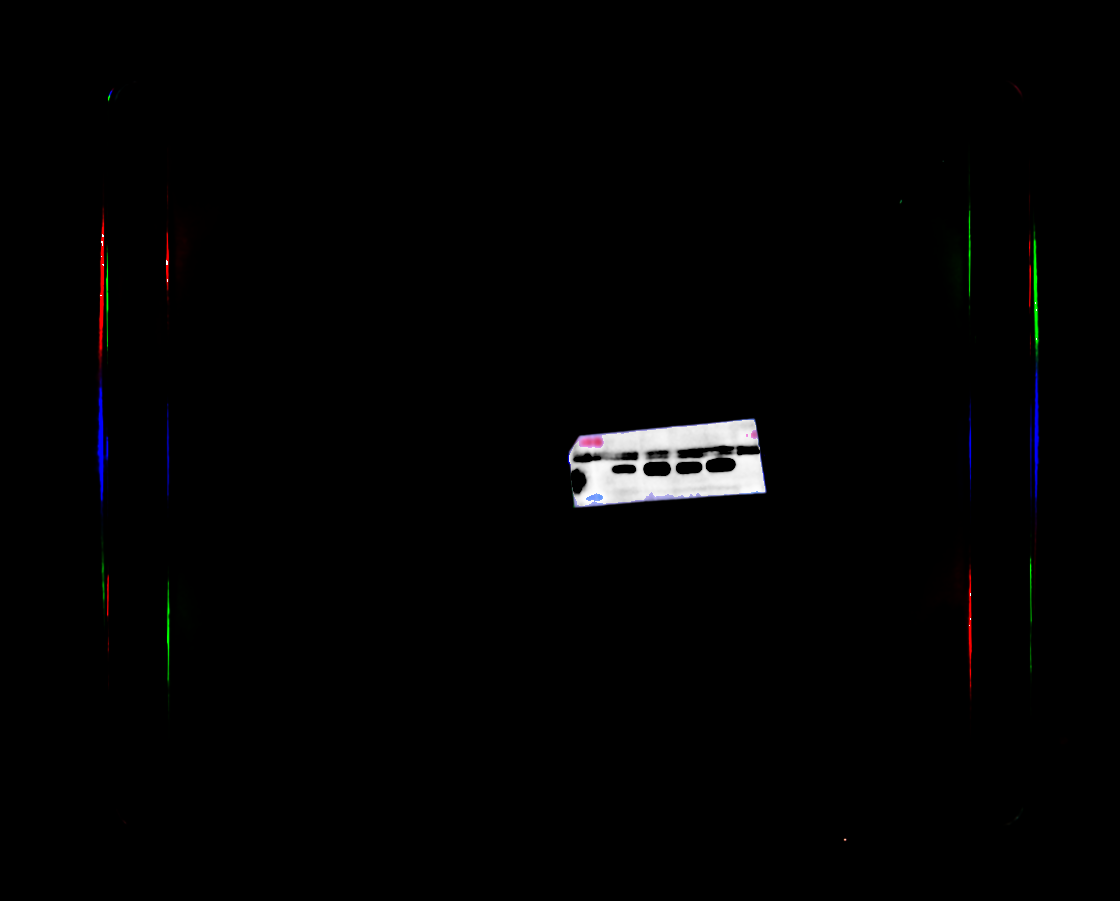

Supplement: Supplementary file 1 [file DataSheet1.zip › WB/P62-4(2).tif]

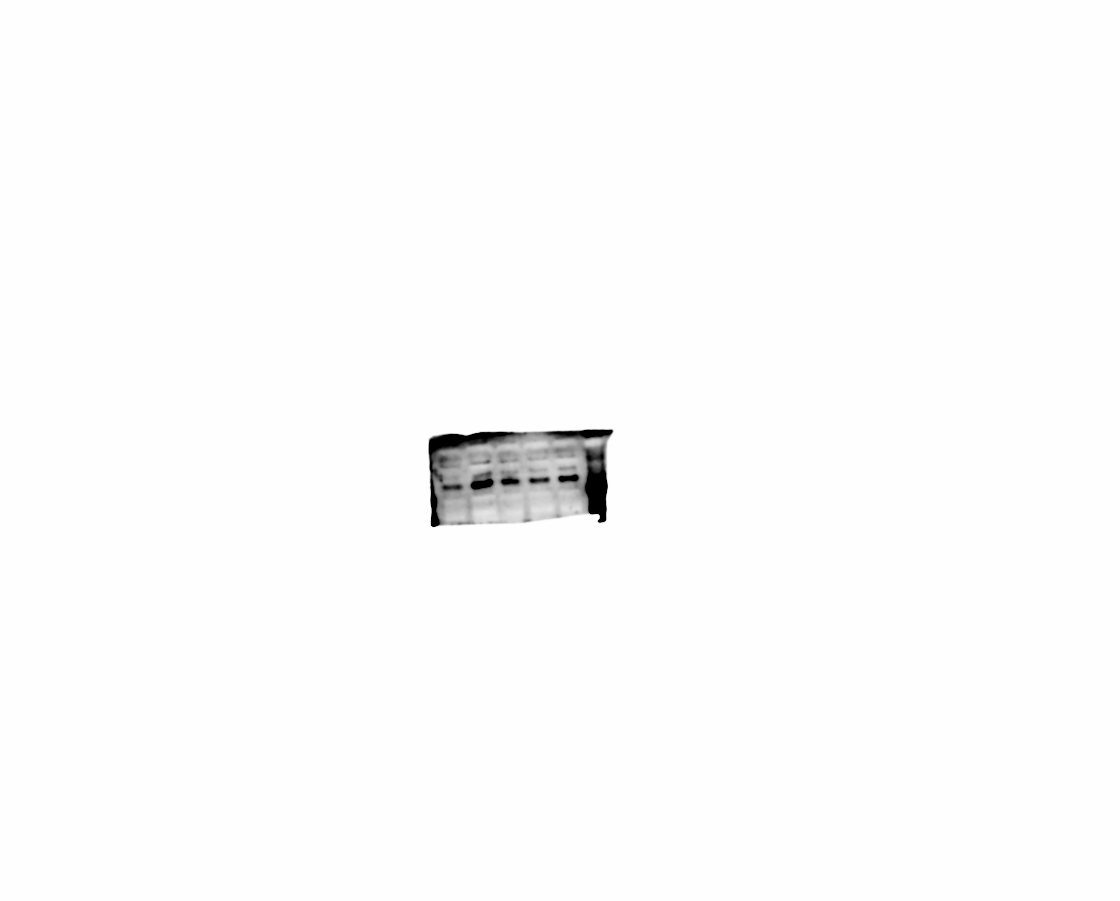

Supplement: Supplementary file 1 [file DataSheet1.zip › WB/P62-5(1).tif]

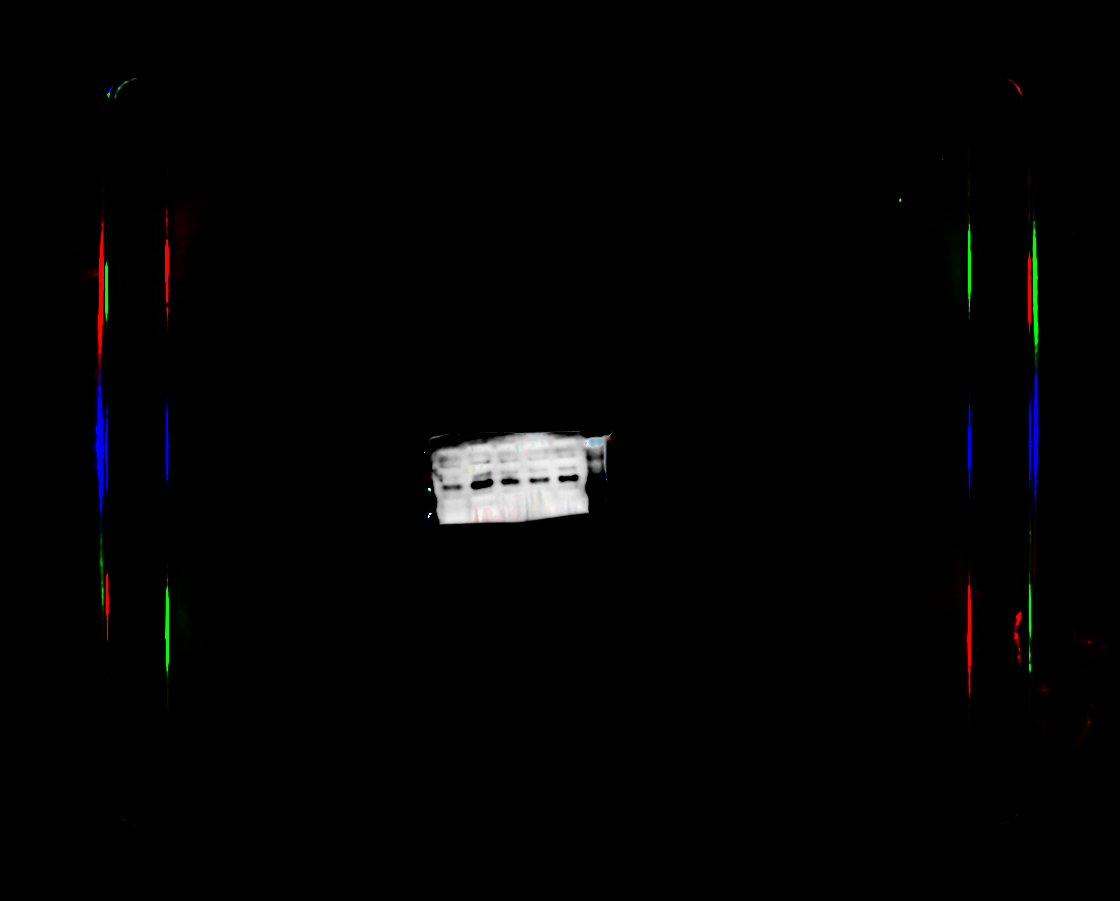

Supplement: Supplementary file 1 [file DataSheet1.zip › WB/P62-5(2).tif]

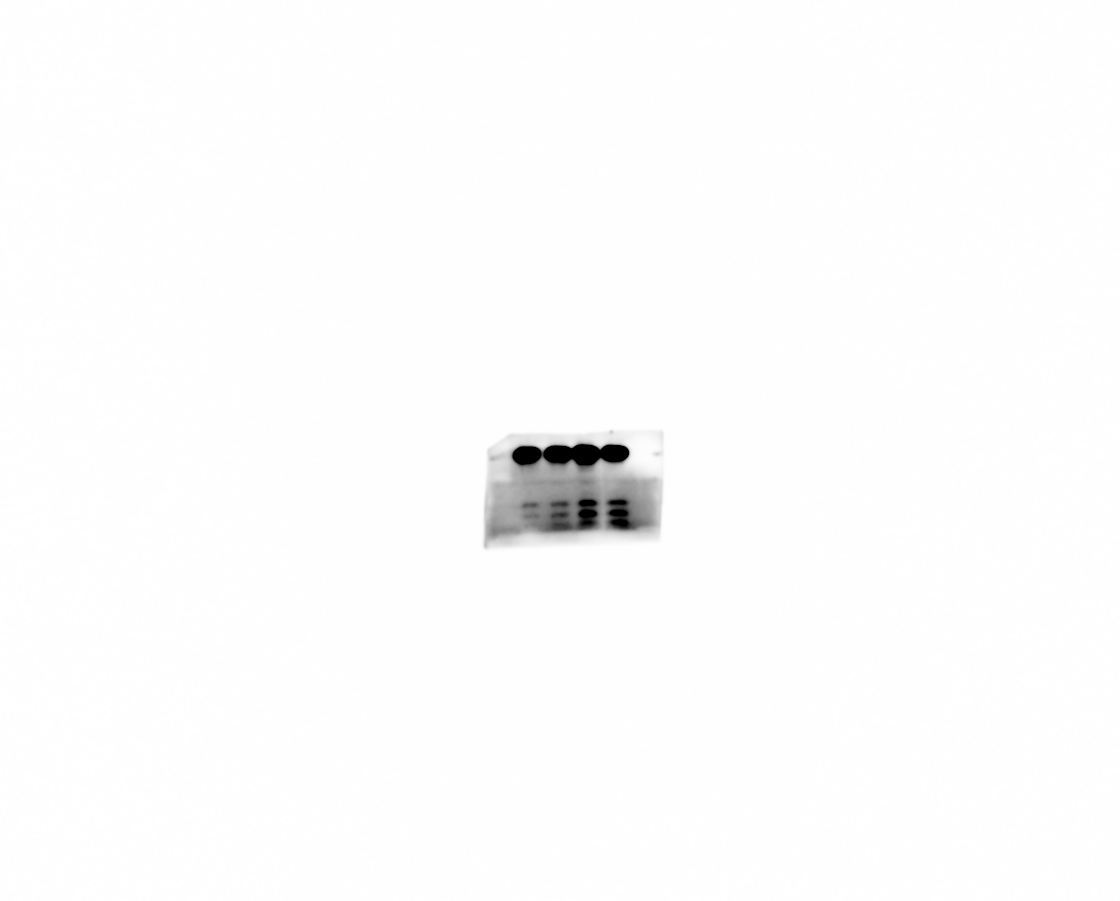

Supplement: Supplementary file 1 [file DataSheet1.zip › WB/RILP-4(1).tif]

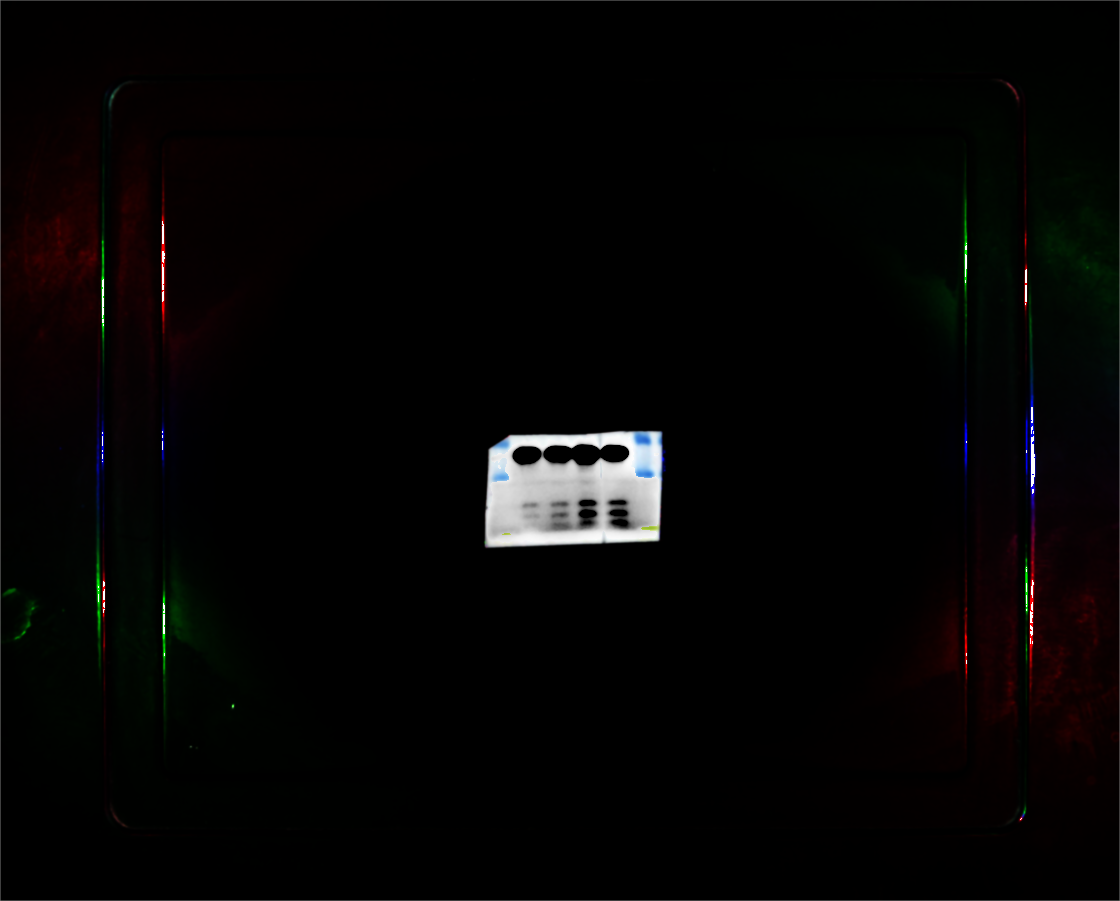

Supplement: Supplementary file 1 [file DataSheet1.zip › WB/RILP-4(2).tif]

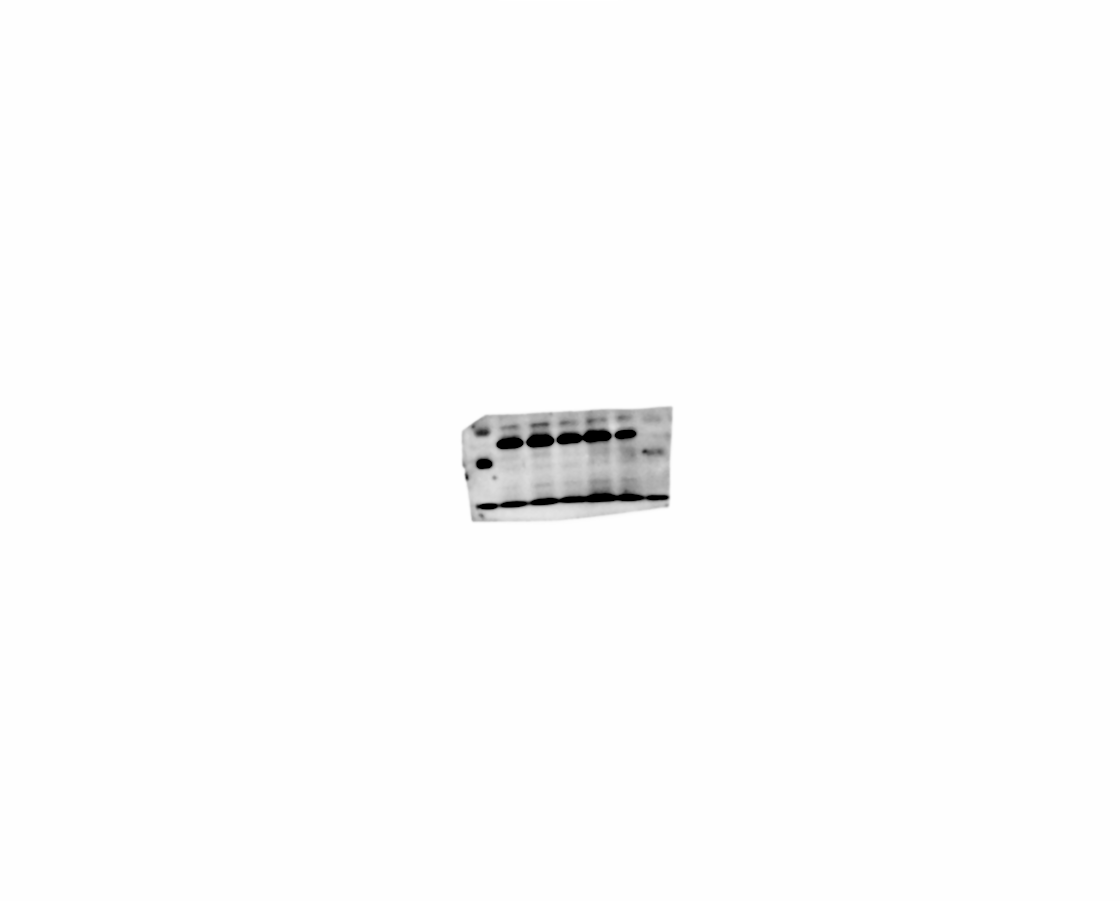

Supplement: Supplementary file 1 [file DataSheet1.zip › WB/RILP-5(1).tif]

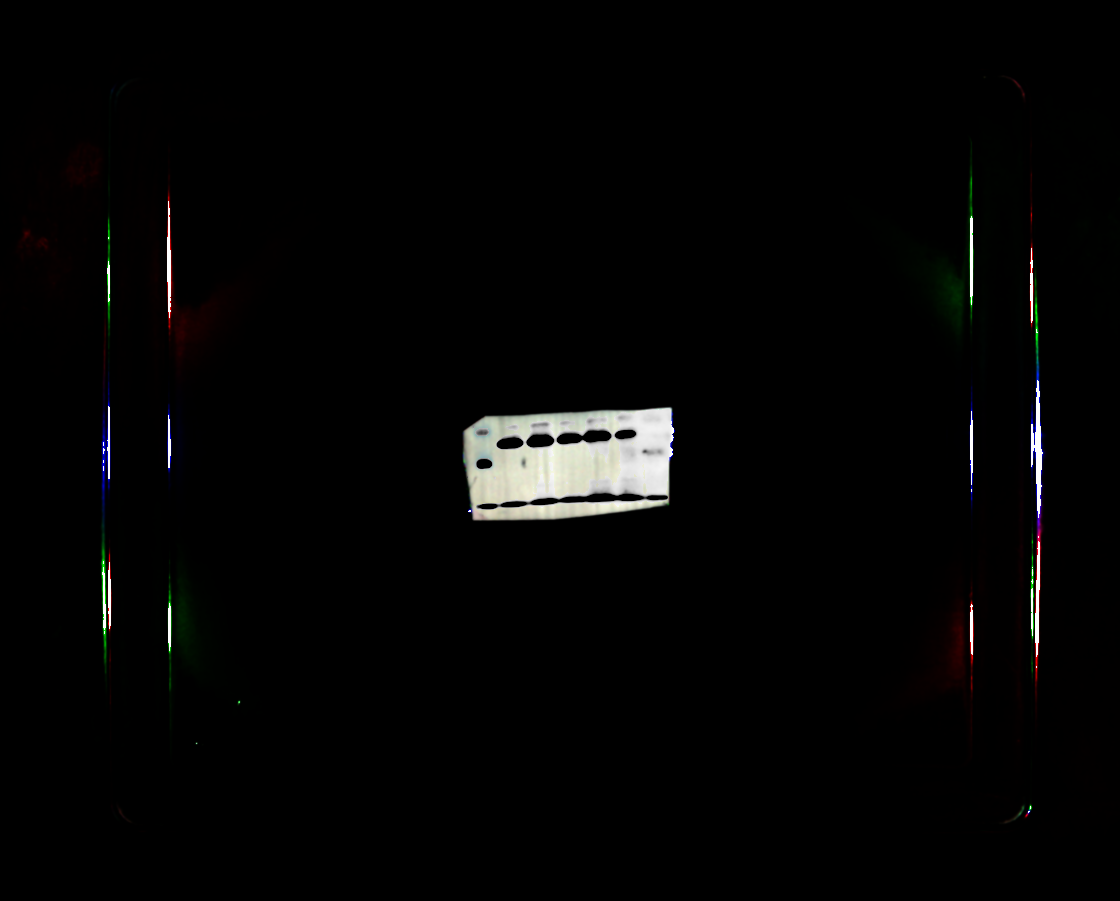

Supplement: Supplementary file 1 [file DataSheet1.zip › WB/RILP-5(2).tif]
